# Improved Discriminative Object Localization Algorithm for Safety Management of Indoor Construction

**DOI:** 10.3390/s23083870

**Published:** 2023-04-10

**Authors:** Jungeun Hwang, Kanghyeok Lee, May Mo Ei Zan, Minseo Jang, Do Hyoung Shin

**Affiliations:** 1 Department of Civil Engineering, Inha University, Incheon 22212, Republic of Korea; 2 Center for Smart Construction Technology, Korea Expressway Corporation, Hwaseong 18489, Republic of Korea; 3 Research Institute of Construction & Environmental System, Inha University, Incheon 22212, Republic of Korea

**Keywords:** object localization, visual explanation, small construction tool, indoor construction site, safety management

## Abstract

Object localization is a sub-field of computer vision-based object recognition technology that identifies object classes and locations. Studies on safety management are still in their infancy, particularly those aimed at lowering occupational fatalities and accidents at indoor construction sites. In comparison to manual procedures, this study suggests an improved discriminative object localization (IDOL) algorithm to aid safety managers with visualization to improve indoor construction site safety management. The IDOL algorithm employs Grad-CAM visualization images from the EfficientNet-B7 classification network to automatically identify internal characteristics pertinent to the set of classes evaluated by the network model without the need for further annotation. To evaluate the performance of the presented algorithm in the study, localization accuracy in 2D coordinates and localization error in 3D coordinates of the IDOL algorithm and YOLOv5 object detection model, a leading object detection method in the current research area, are compared. The comparison findings demonstrate that the IDOL algorithm provides a higher localization accuracy with more precise coordinates than the YOLOv5 model over both 2D images and 3D point cloud coordinates. The results of the study indicate that the IDOL algorithm achieved improved localization performance over the existing YOLOv5 object detection model and, thus, is able to assist with visualization of indoor construction sites in order to enhance safety management.

## 1. Introduction

According to the International Labor Organization (ILO), occupational accidents are disproportionately concentrated in construction industries [1]. The primary cause of fatalities in construction was identified as falls, including tripping and slipping, responsible for over one-third of all fatalities in 2015 [2]. This trend is also observed in the U.S. construction industry, as falls are the main cause of fatalities and injuries [3,4].

Loose materials or objects lying in pathways or falling and striking workers have been identified as major causes of falls at construction sites [5,6]. It is important to remove tripping hazards at construction sites, as protruding objects account for 74.8% of tripping and falling accidents [7].

In 2014, the Occupational Health Agency of the European Union questioned building sites reporting occupational hazards, and its analysis indicated that the majority of organizations have machine or tool risks [8]. To reduce occupational accidents in the construction industry, it is necessary to concentrate on the characteristics of small and medium-sized construction tools and the safety issues associated with them within the indoor construction environment [9]. However, the construction industry has extensive resources concentrated in a limited area, a plethora of data, and a dynamic nature [10], leading to failure in safety management. Since 2000, construction casualties in Korea have increased, indicating that the safety management system is ineffective [11]. Due to the documentation required to comply with the legislation, appropriate safety management, such as on-site safety inspections, is lacking, creating hazardous risk blind spots. Besides, it is impossible to physically examine the whole construction site for worker safety [12]. As the number of indoor construction sites grows, so do concerns about accidents occurring during or after construction [13]. Occupational Safety and Health Administration (OSHA) has also announced plans to extend the Severe Violator Enforcement Program (SVEP) to place a greater emphasis on workplace safety incidents in the construction sector, in response to growing concern about construction safety [14]. Due to the scarcity and imbalance of safety managers’ abilities [15], it is difficult to detect all construction site risk factors in a timely manner.

In order to improve safety management, computer vision-based object recognition technologies are used in the construction safety research area [16]. Computer vision technologies allow for improved management, which may remove construction site dangers and ensure real-time safety.

As a visual aid for safety managers, the scope of construction health and safety research does not fully depict indoor construction site characteristics. Thus, Lee [9] developed a deep learning-based object detection model for indoor construction sites using YOLOv5, which is a cutting-edge object detection technique based on a dataset of 12 commonly used small and medium-sized indoor construction tools. Bounding boxes in the resulting image indicate that object classification and localization were favorable. However, the bounding boxes, a result of the YOLOv5 object detection model, encompass undesirable areas that could not be represented as objects when duplicating and localizing small and medium-sized construction tools for indoor construction site visualization. This may lower the performance of object localization, leading to failure in assisting safety managers in visualizing indoor construction sites.

This study aims to suggest an improved discriminative localization (called “IDOL” in this study) algorithm. The Grad-CAM algorithm employed in the IDOL is designed to accurately mask the regions that deep learning models focus on for object classification, providing a heavily weighted portion of the classification process. The IDOL algorithm demonstrates superior performance over the YOLOv5 object detection model by excluding undesirable noises and thus providing higher localization accuracy. 

Information addressing visual aid for safety management needs accurate object localization. The IDOL algorithm for small and medium-sized equipment is expected to improve the visualization of indoor construction site safety managers and supervisors. Furthermore, the capabilities of the IDOL algorithm extend beyond localization, as it also extracts object coordinates of localization in scanned point cloud data that can be leveraged to create a 3D simulation of construction sites. A 3D visualization of construction sites, with accurate location of construction tools, serves as a valuable visual aid for safety managers, enabling them to manage safety concerns effectively. Consequently, this approach contributes to enhanced safety management on construction sites.

Thus, the IDOL algorithm for small and medium-sized indoor construction tools provides a safer indoor construction site by demonstrating higher localization performance and providing object coordinates of localization compared to YOLOv5 object detection based on 12 classes of small and medium-sized construction tools. The 12 classes of tools in the dataset typify characteristics of indoor construction sites and industry standards.

The IDOL employs gradient weighted class activation mapping (Grad-CAM), which uses heatmaps to clarify classification neural network models by highlighting, or so-called mapping, class activation areas during classification to conduct precise object localization. EfficientNet, the highest performing neural network model in image classification, forms the basis of the IDOL network. EfficientNet-B7, which offered the highest accuracy, was employed for visual explanation during the neural network classification.

Grad-CAM is performed subsequently to train the classification network. Class activation maps visualize where the classification network model is focusing its attention and validate a unique feature of the object in an image in the Grad-CAM model. In the IDOL, this distinguished feature of Grad-CAM is utilized to locate the objects precisely. 

**Figure 1:**
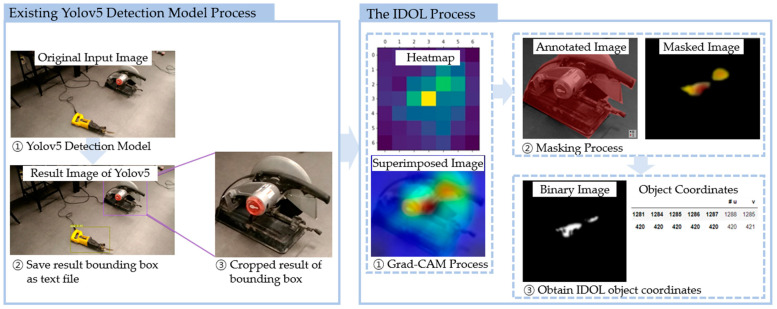
Process of the IDOL algorithm.

The image localization capabilities of Grad-CAM require evaluation to identify whether the class activation map results of the IDOL algorithm are more accurate in object localization than the detected bounding boxes of YOLOv5. Thus, manually segmented images are used as ground truth to assess the localization by an average precision score of the IDOL and YOLOv5 in 2D images via object coordinates (U, V). Furthermore, research findings indicate that the IDOL generates more accurate and efficient localization for indoor construction safety by comparing and evaluating object coordinates (x, y, z) and depth (D) in 3D point cloud mapping for the 3D simulation of an indoor construction environment. Figure 1 depicts the IDOL algorithm process.

## 2. Related Studies

The study aims to develop an improved discriminative localization (IDOL) algorithm for small and medium-sized indoor construction tools to provide visual assistance for safety management. Therefore, the algorithm employs computer vision technologies such as object recognition, image classification, object localization, and visual explanation. In recent studies, computer vision-based object recognition technology has been applied in various construction safety management. 

### 2.1. Computer Vision-Based Object Recognition Technologies in Construction Safety 

Existing safety management is insufficient to decrease occupational deaths and accidents in the construction industry due to its reliance on labor-intensive manual observation, identification, and analysis, which may not deliver immediate and precise hazard information [16]. It becomes increasingly challenging to monitor the whole site and keep track of all workers as construction projects become larger and more complex [12]. Therefore, construction health and safety management must be automated to overcome its limitations [16], and computer vision-based techniques may provide a solution.

In the past decade, machine learning and deep learning-based neural networks in computer vision technologies were established, and computer vision has been applied to mimic and assist human vision. Deep learning-based convolutional neural networks (CNNs) have performed well in object recognition fields [17]. The medical [18] and manufacturing industries [19] have applied deep learning-based object recognition technologies (e.g., image classification, detection, and localization). Compared to other industries, the construction industry has been late in adopting computer vision technology, particularly in the area of construction health and safety. For object recognition to be practical in the construction industry, it necessitates the detection of risk factors at various construction sites. Guo [16] stated that, to conduct comprehensive health and safety monitoring, a thorough understanding of the construction scene at a high level is required and that previous studies on recognizing safety violations in construction sites have to cover all possible safety issues. To that end, construction industries must also address a deficit in the publicly available image database. Kim [13] asserts that preparing training data for neural networks is laborious and time-consuming, hence there exist limitations for individuals and small groups.

Deep learning-based object detection was mainly studied in advance in the construction industry to recognize some restrictive objects and situations [20]. Using an initial deep learning model, studies have been conducted on the recognition of construction equipment. For example, using a region-based fully convolutional network (R-FCN) with transfer learning, Kim et al. [21] demonstrated the detection of heavy equipment such as an excavator, loader, dozer, roller, and backhoe. Azar and McCabe [22] developed a non-rigid equipment detection model based on HOG classifiers to detect objects with articulated shapes, such as hydraulic excavators, in various poses. Yang et al. [23] identified the tower crane jib rotation angle by applying 2D-to-3D rigid pose tracking algorithms. On the other side, studies have also investigated the identification of workers and their safety requirements. Fang et al. [24] employed a two-step method with Faster RNN and deep CNN to monitor workers without harnesses, whereas Park et al. [25] detected workers by utilizing motion, shape, and color cues with image filters and classified them with a k-NN classifier. In addition, Fang et al. [26] and Li et al. [27] each used Faster RNN and YOLOv5 to detect and monitor whether workers wore helmets for safety management.

**Figure 2:**
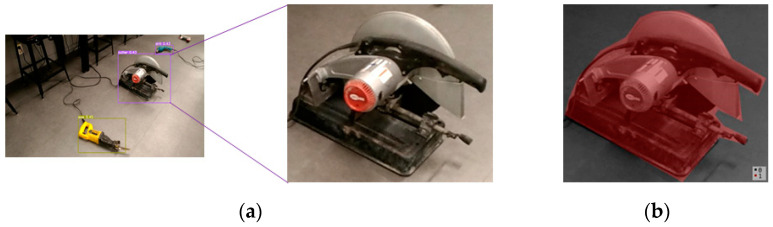
Detection models outcome image and its segmentation. (**a**) Result of YOLOv5 models, (**b**) segmented result image.

Earlier studies focused on workers and safety requirements, as well as heavy outdoor construction equipment. However, object recognition via computer vision for construction safety monitoring remains limited. Management of indoor construction site safety must be a priority to alleviate the current limitation. Detection technologies could replace safety managers in hazardous indoor construction sites to decrease occupational accidents and fatalities. There are many small and medium-sized tools on indoor construction sites that workers may not see, which leads to accidents. Therefore, a deep learning-based object detection model and dataset for 12 small and medium-sized construction tools commonly used on indoor construction sites were built to assist safety managers in visualizing and enhancing construction health and safety using YOLOv5 models [9]. The model represents detection and localization results as bounding boxes in the result images, but the bounding boxes include undesired spaces, which could be defined as noise, and degrade the localization accuracy. Figure 2b displays spaces that are not represented as objects after segmentation by a detection model. 

Furthermore, defining and localizing object coordinates in a 3D point cloud may be hindered by the presence of undesirable spaces in detection results images. For safety managers to utilize it as a visual aid, precise construction object localization information is crucial. Therefore, for more effective indoor construction safety management, a deep learning-based detection model must be developed to reduce the limitations for discriminative localization of small and medium-sized construction tools when simulated as 3D point clouds.

### 2.2. Visual Explanation Technology

In the model of Lee [9], an undesirable space in the bounding box labeled noise necessitated increased object location accuracy in order to precisely correspond object coordinates in 2D to coordinates in 3D point cloud mapping. With more exact object location information derived from 2D images to correspond with 3D point cloud coordinates, safety managers and construction workers are able to reduce the rate of hazardous accidents. Therefore, the IDOL algorithm is proposed to overcome the limitation and to enable practical indoor construction safety management.

“Visual explanation”, also known as “visual interpretation”, has been applied to interpret deep convolutional neural networks by mapping the specific region where the model is giving its attention to classify the object during model learning. It is a promising aid for researchers in comprehending the basis of CNN recognition and is widely applicable in CNN models. 

There are several methods for visual explanation, including activation visualization and feature attribution approaches. However, to utilize the visual explanation method as object localization, class activation mapping (CAM), which falls under the feature attribution method, is appropriate. 

CAM is a type of post hoc visual explanation that enables examining the inside of a CNN and classifying by using the convolution layer responses to perform quantitative object localization [28]. When integrated with the CNN model, CAM calculates the weighted sum of the feature map in a layer and produces an average activation feature map for a predicted class. This map indicates the region where the model focused its attention in order to classify an object and also serves as the localization result.

Zhou [28] evaluated the localization performance of CAM in identifying 200 bird species from the CUB-200-2011 dataset, which contains bounding box annotations using fine-grained recognition. Results confirmed that having a more concentrated image crop enhances discrimination, enabling CAM to accurately localize in image crops and identify object locations with higher precision. 

Nonetheless, CAM has its drawbacks. CAM requires CNN to precede softmax layers immediately; hence, in order to be applied, it must be a very particular type of network structure conducting global average pooling over convolutional maps just before prediction, which may result in performance degradation. In addition, for all other networks, the structure must be modified, and the network must be retrained according to the new architecture. Furthermore, considering that this approach is limited to visualizing only the last convolutional layers of a CNN, it is only effective for analyzing the final stages of the network’s image classification and lacks the ability to provide interpretation of the earlier stages.

Grad-CAM, conversely, is a generalization of CAM that is more adaptable to all CNN structures, yet does not require retraining [29]. Using class-specific gradient information provides a coarse localization map of the important regions in the image with regard to classification, thereby making the model more explainable.

In order to generate Grad-CAM, information on the gradient of the convolutional layer is examined, and the output going through the layer is necessary. With respect to the feature map $A^{k},$ the gradient score for class C, $Y^{c}$ (before the softmax) is calculated. The coordinates of the output passing through the layer to observe ($A^{k_{ij}}$) refers to the activation at position (i, j) of the feature map ($A^{k}$). With these two pieces of information, ($\alpha_{k}^{c}$) is defined, referred to as the neuron importance weight, as follows:

(1) $$\left(\alpha_{k}^{c}\right)=\frac{1}{Z}\sum_{i}\sum_{j}\partial Y^{c}/\partial A_{}^{k_{ij}}$$

Using ($\alpha_{k}^{c}$) and ($A^{k_{ij}}$)), Grad-CAM is finally computed as follows:

(2) $$\left(L_{Grad-CAM}^{c}\right)=ReLU(\sum_{k}\alpha_{k}^{c}A_{}^{k_{ij}})$$

**Figure 3:**
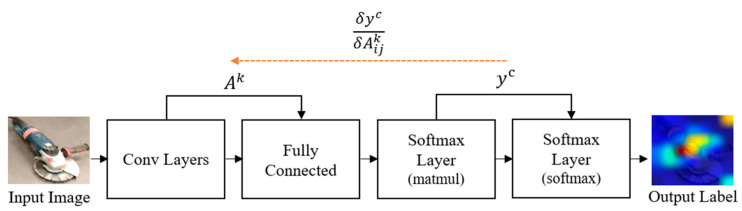
Grad-CAM architecture.

It is mentioned that the final use of ReLU is due to its interest in only the positive features of a class. In addition, when ReLU was experimentally eliminated, the localization performance declined substantially. Figure 3 demonstrates the model architecture of Grad-CAM. Grad-CAM offers an efficient and adaptable method of visualizing the important areas of an image in a CNN, making it more explainable and interpretable.

Various fields, such as medical industries, e.g., [30,31], gain an advantage from object localization using class activation maps. For post hoc interpretability of classification CNNs, saliency maps, feature attribution maps, and class attribution maps have grown in popularity. Medical imaging necessitates discriminative localization of objects as numerous deep learning medical imaging studies use saliency maps to validate model prediction and localize medical images [32].

Thus, class activation maps derived from a method such as Grad-CAM, could serve as a localization algorithm for improved discriminative localization of objects in images that are more advanced than YOLOv5 [33] detection models by minimizing undesirable, noisy areas contained in the resulting bounding boxes. Grad-CAM is utilized for identifying specific points or features of an object that deep learning models use to make classifications. The IDOL algorithm for small and medium-sized construction tools is able to reduce occupational accidents by providing safety managers and supervisors enhanced visualization by deriving accurate localization of the construction tools.

## 3. Improved Discriminative Object Localization (IDOL) Algorithm

The IDOL algorithm utilizes detected bounding boxes from YOLOv5 object detection model [9], using the dataset of small and medium-sized indoor construction tools. Then, images are cropped according to the bounding box, which are then processed by the IDOL algorithm. In the process, cropped images are used as input for Grad-CAM, which employs EfficientNet-B7 as a base classification network model, to obtain localization results. 

### 3.1. YOLOv5 Detection Algorithm

**Figure 4:**
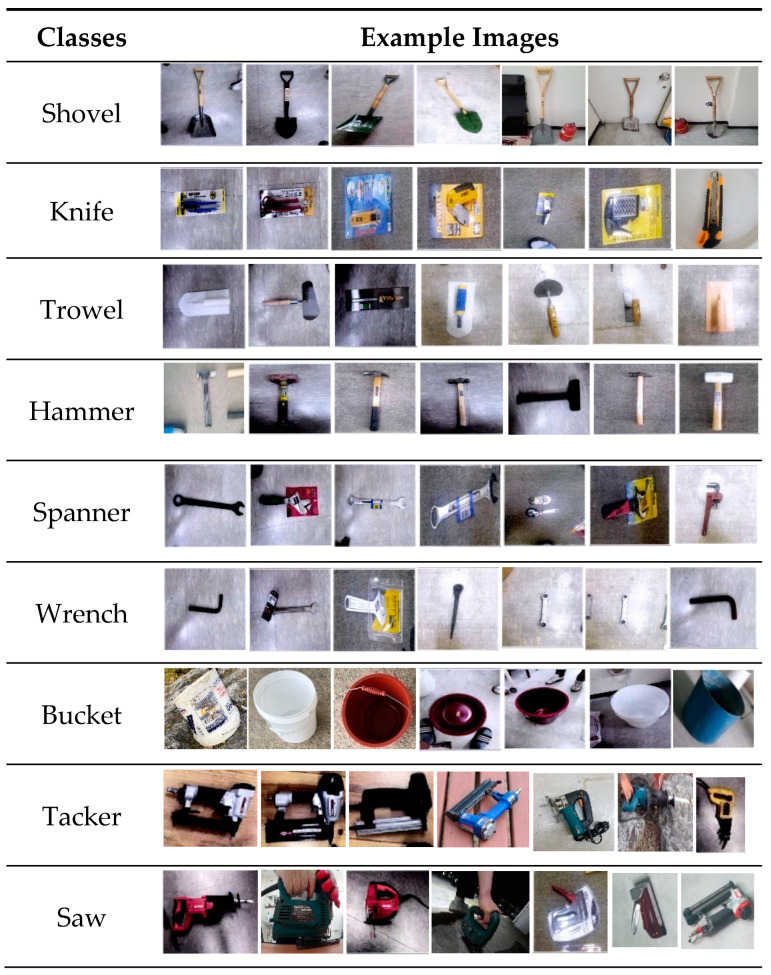

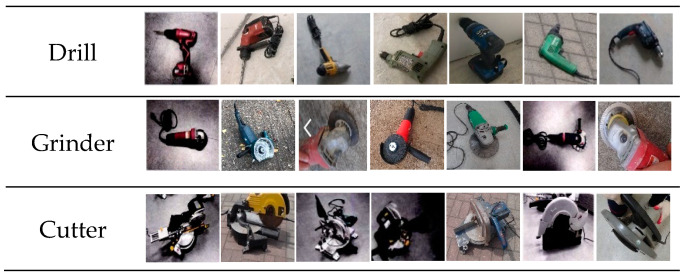
Examples of small and medium-sized indoor construction tools dataset [9]. (Reproduced with permission from Lee et al., KSCE Journal of Civil Engineering; published by Korean Society of Civil Engineers, 2023).

YOLOv5 object detection model is based on a single-stage detector using deep learning, designed for detecting small to medium-sized tools on indoor construction sites [9]. To collect data for the development of YOLOv5 models, a dataset of 48,477 images representing 12 small and medium-sized construction tools commonly used across many indoor construction projects was selected as displayed in Figure 4. The study utilized the YOLOv5s algorithm, which has the fastest detection rate, and YOLOv5x, which has the highest accuracy in the field of real-time object recognition, as shown in Table 1.

The limitations of undesirable areas which are not defined as objects contained in detected bounding boxes, leading to the degradation of localization performance, still remained. Due to the constantly changing characteristics of construction sites, the situation that poses a threat to the safety of workers in the same place may occasionally fluctuate, necessitating the use of real-time results to conduct safety management efficiently. Thus, the IDOL algorithm ensures that object localization on the cropped bounding boxes of detection results is more discriminative and effective. When identifying and matching the localization information of an object to a 3D point cloud coordinate, the location of an object in the image should be accurately localized. The implementation of the IDOL is expected to serve as a visual aid for indoor construction site safety managers through improved object localization of small and medium-sized construction tools.

### 3.2. EfficientNet-B7 Image Classification Network

Convolutional neural networks (CNNs) have grown significantly and have produced outstanding results since LeCun et al. [34] first presented them as a unique neural network model. The primary objective of image classification is to comprehend the entire image as a whole, with the focus being to label the image to facilitate classification specifically. 

In this study, image classification serves as a precursor to gradient weighted class activation mapping (Grad-CAM). To generate optimal learning results, a neural network for image classification was developed based on EfficientNet-B7. Among deep learning-based neural networks, EfficientNet is more efficient than ResNet-101 and DenseNet-201, which are pre-trained on the ImageNet dataset and reach a state-of-the-art 84.3% accuracy [27]. The ImageNet dataset contains 14,197,122 images, which are publicly available training images and manually annotated.

Transfer learning, a method of learning that involves transferring a neural network trained in the past using different image data to a new neural network, is employed in this study. This method offers the benefits of reduced learning time and minimal processing. Loading and training an EfficientNet-B7 network architecture that is pre-trained using ImageNet weights resulted in much better training precision and loss than initializing and training from scratch.

#### 3.2.1. Training Data and Preprocessing

To facilitate the deep learning-based YOLOv5 object detection model, a high-resolution dataset featuring small and medium-sized construction tools was generated in a previous study, encompassing a multitude of shooting locations and obstacles to recognition such as backdrops, angles, luminance, occlusion, and more [9]. Twelve types of small and medium-sized construction tools (e.g., bucket, cutter, drill, grinder, hammer, knife, saw, spanner, shovel, tacker, trowel, and wrench) commonly used in indoor construction sites were selected as classes of the dataset. A dataset of 33,205 still images is used for the image classification process of this study for small to medium-sized construction tools. A total of 60% of the constructed image data was distributed for training, 20% for validation, and 20% for testing.

Before being fed into the neural network model, raw image data must undergo a preprocessing stage to reduce unintended modifications by resizing to meet the input dimension requirements and normalizing for a better visualization, and amplifying image attributes pertinent to the subsequent classification task. To this end, image augmentation has been implemented in this study, exposing the model to a diverse range of training data and mitigating the risk of overfitting during the classification process.

#### 3.2.2. Model Architecture and Compiling

CNNs are capable of improving classification accuracy by scaling deep layers and their complex features. Tan and Le [35] have demonstrated that scaling neural network dimensions such as width, depth, or resolution are able to enhance accuracy significantly. To achieve optimal accuracy and efficiency, the scaling of CNN must balance network width, depth, and resolution.

The scaled dimensions of the network are interdependent, and Tan and Le [35] have proposed a compound scaling method that uses a compound coefficient of (φ) to proportionally scale width, depth, and resolution as in the following Equations (3) and (4).

(3) $${max}_{d;w;r}Accuracy\left(N\left(d,w,r\right)\right)\;s.t.N\left(d,w,r\right)={⊙}_{i=1\ldots s}\hat{F^{d\hat{{.L}_{i}}}}(X_{<\gamma .\hat{H_{i}}\gamma .\hat{W_{i}}\gamma .\hat{C_{i}}>})$$

(φ) specifies how many resources are available for model scaling, whereas (α, β, γ) describes how to allocate these extra resources to network depth, width, and resolution to balance the dimension. The following Equation (4) shows that convolutional operations consume floating point operations per second (FLOPS) proportional to d, ($w^{2}$), and ($r^{2}$). ($\alpha \cdot \beta^{2}\cdot \gamma^{2}\approx 2$) to 4 is constrained so that for every new (φ), the required FLOPs increase by ($2^{\varphi })$.

(4) $$Memory\left(N\right)\leq target\;memory;\;FLOPS\left(N\right)\leq target\;flop\;depth:d=\left(\alpha^{\varphi }\right),width:w=\left(\beta^{\varphi }\right),resolution:r=\left(\gamma^{\varphi }\right)\;s.t.\alpha \cdot \beta^{2}\cdot \gamma^{2}\approx 2(\alpha \geq 1,\beta \geq 1,\gamma \geq 1)$$

The baseline network architecture must have sufficient accuracy for further improvements as model scaling relies on baseline architectural configuration. EfficientNet-B0 was designed using MnasNet’s search space method [36]. First, (φ) is initially fixed to 1, assuming twice the amount of resources, then a small grid search of (α, β, γ) based on Equations 3 and 4 is undertaken. As a result, the optimum values for EfficientNet-B0 are (α = 1.2), (β = 1.1), (γ = 1.15) under constraint ($\alpha \cdot \beta^{2}\cdot \gamma^{2}\approx 2$). Then, (α, β, γ) is fixed, and as in Equation (3), the baseline network with varied (φ) is scaled up. All EfficientNet models, including EfficientNet-B7 employed in this study as a base classification network model, are scaled likewise from the baseline model EfficientNet-B0 [35]. 

#### 3.2.3. Training and Evaluating Accuracy

The first step for transfer learning is to freeze all layers and train only the top layers. A relatively large learning rate (1 × 10^−2^) is used for the first step. Validation accuracy and loss will usually be better than training accuracy and loss. This is because the regularization is strong, which only suppresses training time metrics. The convergence may take up to 50 epochs, depending on the choice of learning rate. If image augmentation layers were not applied, the validation accuracy would only reach 74.09%. 

**Figure 5:**
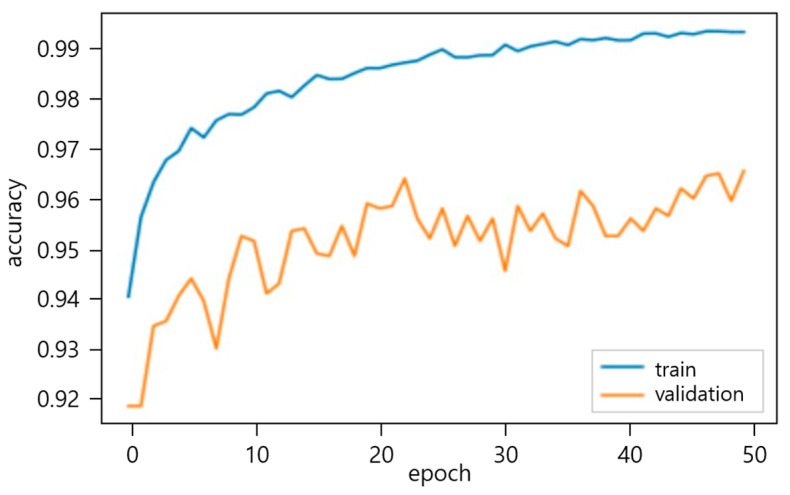
EfficientNet-B7 model classification accuracy.

The experimental results of the high-resolution image dataset of small and medium-sized construction tools have demonstrated a remarkable accuracy of 95.56% for the optimal neural network, as shown in Figure 5. This outcome indicates a significant improvement of 21.47% over the accuracy of 74.09% achieved before fine-tuning. This finding is valuable for developing a highly efficient object detection algorithm that targets small to medium-sized tools on construction sites. The fine-tuning process involved a layer-by-layer re-learning of the transferred EfficientNet-B7, followed by selecting the neural network with the highest performance as the optimal model. Employing the Grad-CAM technique for object localization in the image classification process may further enhance the accuracy of the IDOL network model by providing precise and robust visual explanations by generating class activation maps.

### 3.3. Localization by Gradient Weighted Class Activation Mapping (Grad-CAM)

To apply the Grad-CAM algorithm, importing several open-source software libraries such as Tensorflow, Keras, and OpenCV are required to provide a Python interface for neural networks. The pre-trained EfficientNet-B7 model, an image classification neural network, is employed for the small and medium-sized construction tools dataset by transfer learning. Due to the input dimension requirement of (224 × 224 × 3) for the EfficientNet-B7 network, image resizing is necessary, and normalization of the input image between 0 and 1 is done by using the preprocess_input function for visualization. The “top activation”, the output from the final convolutional layer of the EfficientNet-B7 model, and the class activation map for the image’s top prediction are obtained for the results. 

**Figure 6:**
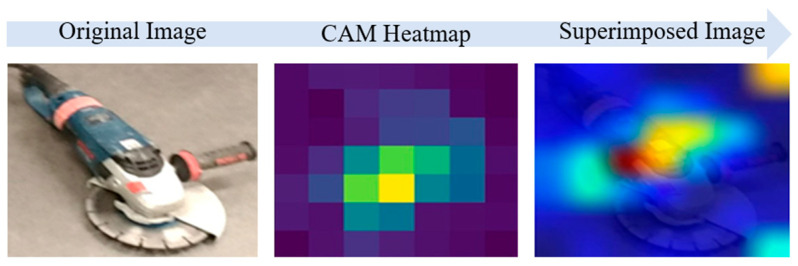
Visualization of Grad-CAM heatmap and the superimposed image.

The Grad-CAM generates a feature map that is 7 × 7 × 512 in size, where the gradient of the class output value with respect to the feature map is derived by combining the gradients across all axes except the channel dimension. The feature map is then transformed into a heatmap by assigning weights to the computed gradient values. The generated heatmap is then scaled and superimposed on the original image using OpenCV to visually represent the regions where the EfficientNet-B7 model focuses its attention on classification. An example of a Grad-CAM heatmap and superimposed image is displayed in Figure 6. 

The Grad-CAM is advantageous for discriminative object localization [29], and essential in the research procedure for small and medium-sized construction tools. The highlighted regions mapped in red and orange colors on the heatmap, and superimposed images provide insights into where the classification model is focusing its attention to differentiate between classes, leading the algorithm to leave out coordinates in unwanted areas and only use selected coordinates in highlighted red and orange regions which represent the location of an object with higher accuracy. Therefore, to assess the localization performance of the IDOL algorithm, the highlighted regions are masked according to the color space areas and pixel coordinates obtained.

## 4. Evaluating Localization Accuracy of 2D Object Coordinates

The IDOL algorithm utilizes Grad-CAM to achieve accurate object localization not only in 2D images but also in 3D point cloud simulation to enhance indoor construction site visualization. Performance and accuracy of small and medium-sized construction tool localization must be assessed by an evaluation of the object coordinate (U, V) results of YOLOv5 and the IDOL with ground truth segmentation results. 

### 4.1. Indoor Construction Site Simulation

An indoor construction site is arranged for the simulation with 24 small and medium-sized construction tools. Each class is represented with two models, which are placed at random in the experimental area to imitate an indoor construction site, and 3D point cloud mapping of the simulated site is carried out afterward. The device used for 3D point cloud mapping is Lenovo Phab 2, a mobile device from Google Project Tango capable of detecting motion, depth, and area. The still images and videos are set and shot in 12 million pixels, at a pixel density of 0.0352 cm, with a focal length of 35 mm.

**Figure 7:**
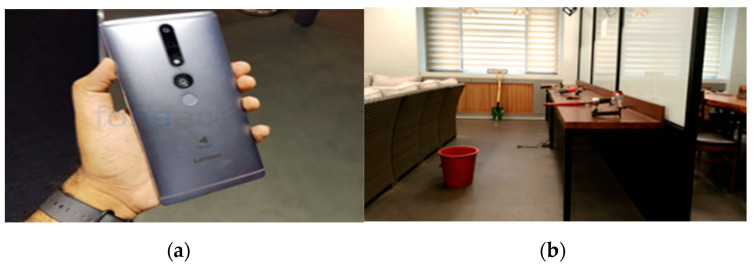
Simulation utilities for experimental data, (**a**) simulated indoor construction environment, (**b**) device used for 3D point cloud mapping.

**Figure 8:**
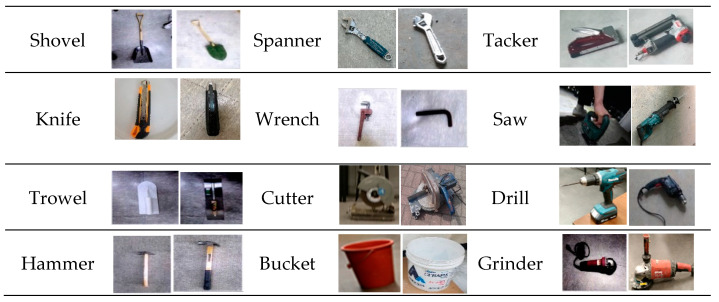
Models of small and medium-sized construction tools.

A total of 192 images were collected from the simulated site for evaluation of object coordinates. These images depicted various angles of objects from different positions and were used to compare the performance of YOLOv5 object detection and the IDOL algorithm with ground truth segmentation. The purpose was to test and assess the localization performance of each approach. Figure 7a,b and Figure 8 show the simulated indoor construction environment, 3D point cloud mapping device, and 24 models of small and medium-sized construction tools used, respectively.

### 4.2. Experiment Data Preparation

The IDOL algorithm employs Grad-CAM to accurately localize an object in images and 3D point cloud to enhance indoor construction site visualization. The localization performance needs to be identified by the evaluation of object coordinate results from the IDOL and YOLOv5 detection. Accuracies of each algorithm are then calculated using average precision score by considering the segmented ground truth coordinates of image data.

#### 4.2.1. IDOL Algorithm Results

**Figure 9:**
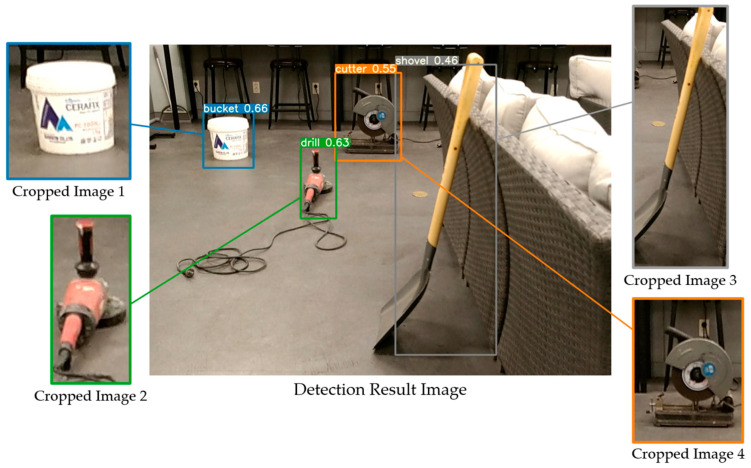
Result of cropped bounding boxes in an image.

The IDOL algorithm is a deep learning-based localization approach for small and medium-sized construction tools. The algorithm utilizes prior YOLOv5 object detection results to crop and refine the bounding boxes to accurately localize the object in the image, as demonstrated in Figure 9.

**Figure 10:**
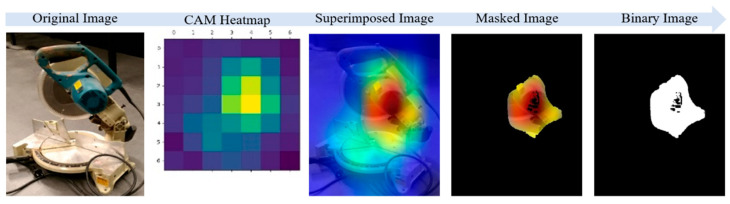
Original and the IDOL algorithm result images.

To improve the accuracy of object localization in the images, the cropped images are classified using a Grad-CAM process based on EfficientNet-B7 neural network, which then generates a class activation map highlighting the area where the classification network identifies the object. The superimposition of the heatmap on the original image facilitates the visualization of the class activation results, with different color spaces integrated, including red, orange, yellow, green, and blue. The red and orange areas of the color spaces serve as highlights on the class activation map, indicating that the classification network focuses primarily on these areas as objects. The red and orange areas are then masked to retrieve the coordinates of pixels for object localization in the masked image. The masked area of the image is converted to a binary image, and the resulting matrix provides more pinpointed object localization coordinates (U, V). An example of a cutter image throughout the above process is displayed in Figure 10. 

#### 4.2.2. YOLOv5 Detection Results

**Figure 11:**
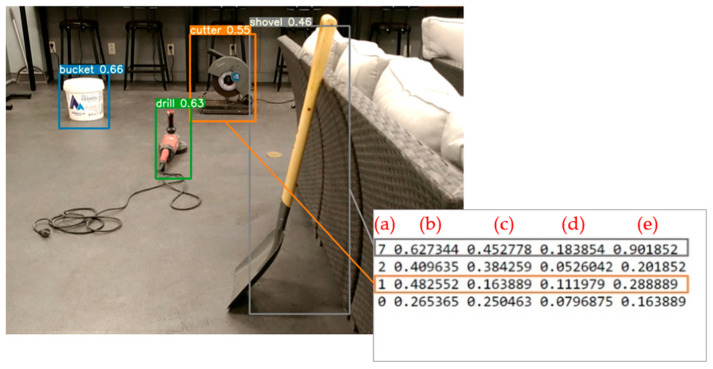
Sample result images and corresponding result text file.

In a previous study [9] on a YOLOv5 object detection model, training two models resulted in favorable performance. Specifically, the M1 and M2 models trained with YOLOv5s achieved mAP@0.5 scores of 69.1% and 64.4%, respectively. Subsequently, a total of 192 images were collected to evaluate the presented algorithm. The M1 model, which exhibited superior detection accuracy for small and medium-sized construction tools, was employed to recognize the 192 experimental images and generate bounding boxes for the experimental data as text files. The class information for each object detected by the M1 model, including (a) object class ID, (b) horizontal center pixel coordinates of the labeling box (U), (c) vertical center pixel coordinates (V), (d) horizontal pixel length of the box (W), and (e) vertical pixel length (H), are presented on the right side of Figure 11. The object coordinates (U, V) of the detected bounding boxes were transformed into 3D point cloud coordinates and compared with the results of the IDOL algorithm. 

#### 4.2.3. Object Coordinates of Ground Truth Segmentation

**Figure 12:**
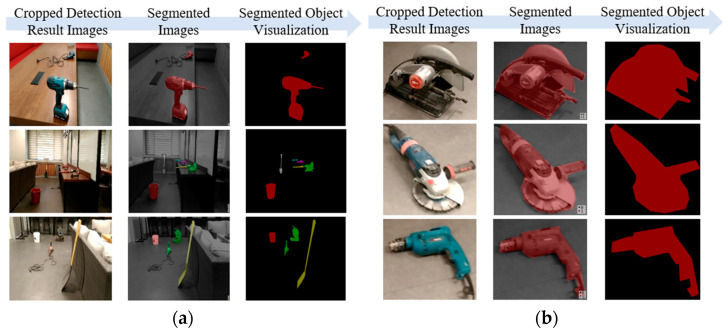
Visualization example of segmented images by ‘Labelme’, (**a**) models of small and medium-sized construction tools, (**b**) simulated indoor construction environment.

The evaluation process involves comparing the object coordinates each generated by the IDOL algorithm and the YOLOv5 object detection model, with manually segmented images as the ground truth. After completion of the YOLOv5 process, the detected bounding box areas are cropped to facilitate the following process of the IDOL algorithm. The cropped images are manually segmented via ‘Labelme’ [37]. Furthermore, the original input image is also segmented to enable an object coordinate to correspond with 3D point cloud data. The segmented images and annotation text files serve as ground truth for qualitative and quantitative comparisons of localization efficiency. ‘Labelme’ is a Python-based graphical image annotation tool that facilitates the construction of datasets, for instance, segmentation, semantic segmentation, bounding box detection, and classification, and was inspired by MIT. The ‘Labelme’ is installed in the ‘Anaconda’ environment, and the segmented and annotated images are converted into a Pascal VOC instance segmentation dataset. As the study focuses on comparing pixel coordinate information, annotation of the training images is necessary. An example of a segmented image containing 12 small and medium-sized construction tool classes is shown in Figure 12.

### 4.3. 2D Object Coordinates Evaluation

In the following sections, object coordinates (U, V) of 2D images are evaluated. A safety manager must be able to depend on the localization results to establish the coordinates of indoor construction tools in 2D images. Thus, localization capabilities per the new localization method are evaluated to obtain a high level of localization performance, which requires the accurate recognition of object class and location. Thus, the localization capacity assessment computes the average precision score and accuracy qualitatively and quantitatively.

#### 4.3.1. Average Precision Score

**Figure 13:**
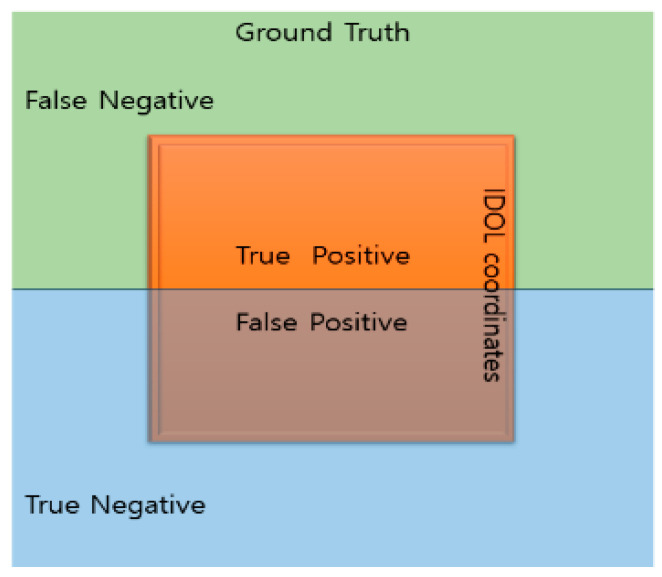
Confusion matrix for object localization results.

“Precision” is defined by a confusion matrix between the ground truth (GT) and the resultant object coordinates as the correct or incorrect object localization. Precision quantifies the accuracy of the coordinate results, and the proportion of accurate object coordinates in GT for the current study. Figure 13 depicts a confusion matrix based on GT and the IDOL coordinates.

Precision is the ratio of true positives (TP) to predicted positives (TP+FP), as demonstrated by Equation (5). The coordinates inside the ground truth segmentation are regarded as positive labels to determine the intersection between the IDOL coordinate results of masked image and ground truth segmentation. As true negative comprises the majority of the image, it is less significant for the metric. The confusion matrix determines the proportion of correct IDOL object coordinate results. Thus, the precision score may be calculated using all object coordinates in the masked image, with 1 being the highest accuracy score and 0 the lowest.

(5) $$Presicion=\frac{TP}{TP+FP}=\frac{TP}{All\;Detections}$$

**Figure 14:**
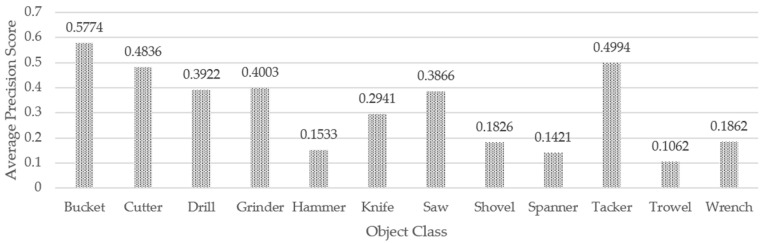
Average precision score for YOLOv5 object coordinates.

The average precision of the IDOL coordinates (U, V) for small and medium-sized interior construction tools is 1, indicating that they are regarded as true positive of ground truth coordinates. YOLOv5 detection has an average accuracy of 0.317 across all tool classes. The bucket has the highest average accuracy score (0.577) among 12 classes of indoor construction tools, while the trowel has the lowest (0.106). The average accuracy score for YOLOv5 object detection model over 12 types of indoor construction tools is displayed in Figure 14.

#### 4.3.2. Evaluation of Object Localization Accuracy

The average precision score demonstrates that the IDOL object coordinates consistently persist in the ground truth (GT) object coordinates. Accordingly, the total number of object coordinates in GT is already specified when calculating the accuracy of the localization utility. The IDOL localization accuracy is calculated by multiplying the precision value of the IDOL based on GT by the number of object coordinates in the IDOL ($N_{IDOL}$) that belong to GT and are divided by the total number of object coordinates in the IDOL as described in Equation (6). 

Then, YOLOv5 detection bounding box accuracy is calculated by the number of YOLOv5 object coordinates ($N_{Yolov5}$) in GT divided by the total object coordinates of the YOLOv5 detected bounding box, as described in Equation (7). The total number of YOLOv5 object coordinates is obtained from the YOLOv5 detection result text file, which includes the size of the bounding box, since YOLOv5 detection coordinates are equal to the product of the bounding box width and height. Subtracting the total number of GT coordinates ${(N}_{GT}$) from ($N_{Yolov5}$) yields ($N_{Yolov5\in GT}$) as in Equation (8).

(6) $$Accuracy_{IDOL}=\frac{N_{IDOL\in GT}}{N_{IDOL}}\times 100$$

(7) $$Accuracy_{Yolov5}=\frac{N_{Yolov5\in GT}}{N_{Yolov5}}\times 100$$

(8) $$where,\left(N_{Yolov5\in GT}\right)=N_{Yolov5}-N_{GT}$$

**Figure 15:**
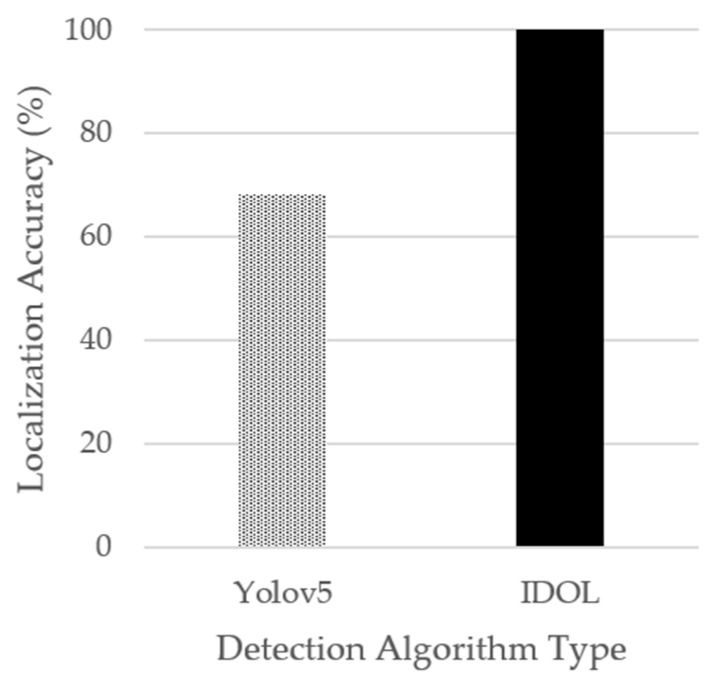
Object coordinates (U, V) accuracies.

Figure 15 analyzes the 2D object coordinates (U, V) of the IDOL and YOLOv5 detection algorithms based on GT coordinates to demonstrate their corresponding object localization accuracy. The IDOL’s object localization is superior to that of YOLOv5 in terms of localization accuracy. Additionally, the utilization of the IDOL effectively reduces the unwanted spaces included within the YOLOv5 detection result bounding boxes to gain more accurate result coordinates for object localization. 

## 5. Evaluation of Localization Accuracy of 3D Object Coordinates

The IDOL algorithm generates 3D object coordinates of localization results to integrate the object localization information into a simulation of a construction site for 3D visualization, providing an enhanced visual aid for safety managers. In this study, the performance of the proposed algorithm to obtain 3D coordinates of localized objects is verified through an experiment in an simulated indoor construction site. The performance is evaluated by comparing (x, y, z) coordinates of 3D point clouds and depth information. The algorithm has demonstrated superior localization performance by comparing the object coordinates (U, V) in 2D images. 

This study proposes the practical implementation of the IDOL algorithm as a visual aid technology for safety management at indoor construction sites. This is to serve as a visual aid to improve safety, because localizing object solely by resulting 2D coordinates is insufficient. For efficient safety management, detecting the distance between the construction workers and tools is essential, which can be obtained by the depth in 3D point cloud data. 

The output of the IDOL must be evaluated in 3D coordinates with depth information by matching 2D object coordinates with the 3D point cloud coordinates to calculate the depth on indoor construction sites. For empirical evaluation, a comparison of object coordinates (U, V) with their corresponding (x, y, z) 3D point cloud coordinates and depth values (D) must be considered along with object localization errors. 

### 5.1. Preparing 3D Point Cloud Data

**Figure 16:**
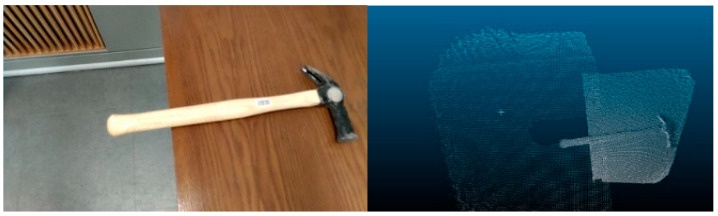
Object RGB image data and collected 3D point cloud data.

For safety managers to comprehensively examine indoor construction conditions, twinning a simulation environment is essential. The twining process involves using a pro smartphone to conduct 3D mapping, which produces RGB images and point cloud data. The resulting simulated environment is an accurate replica of the real scene, with an RGB image pixel size of 1920 × 1080. For the experiment, simulated indoor construction sites were set, and Figure 16 provides an example of data visualization for a simulated environment. The depth values are calculated using Equation 9, and the 3D point cloud data structure includes (x, y, z) coordinates, the corresponding 2D coordinates (U, V), and the depth value (D), as outlined in detail in Table 2. The depth values (D) and 3D point cloud coordinates (x, y, z, U, V) are used to match and compare object coordinates in 2D images (U, V). The corresponding (x, y, z) and (D) values of the ground truth (GT), the IDOL, and YOLOv5 algorithms are used to calculate the object localization errors that may occur during 3D point cloud mapping.

(9) $$Depth=\sqrt{\left(x\times x\right)+\left(y\times y\right)+\left(z\times z\right)}$$

### 5.2. Evaluation in 3D Point Cloud Coordinate (x, y, z) and Depth (D)

The objective of the evaluation is to demonstrate that the proposed IDOL algorithm outperforms the existing YOLOv5-based detection algorithm and is more reliable in terms of increased localization capabilities. The initial step involves matching the 2D object coordinates (U, V) of GT, IDOL, and YOLOv5 with the (U, V) coordinates of the 3D point cloud. The corresponding (x, y, z) and depth (D) values are then obtained. According to the matching data, there are several (x, y, z) and (D) values that reflect the 2D object coordinates; thus, it is necessary to estimate the mean value of (x, y, z) coordinates and (D) for each GT, IDOL, and YOLOv5. The mean (x, y, z) coordinate values define the location of objects in a 3D point cloud.

To calculate the mean value of a set of (x, y, z) and depth (D), the mean values of (x, y, z) and (D) are calculated, followed by the calculating differences between GT and the IDOL, and between GT and YOLOv5. As the ground truth (GT) represents the true coordinates of the object location when 2D object coordinates are mapped onto a 3D point cloud, the differences between the GT and the suggested algorithms are referred to as localization errors. The localization errors for each type of indoor construction tool are calculated using the following Equations (10) and (11).

(10) $${Localization\;Error}_{IDOL}={mean(x,y,z,D)}_{GT}-{mean(x,y,z,D)}_{IDOL}$$

(11) $${Localization\;Error}_{Yolov5}={mean(x,y,z,D)}_{GT}-{mean(x,y,z,D)}_{Yolov5}$$

The localization errors of small and medium-sized indoor construction tools are presented in Table 3. Table 3 depicts the localization error in 3D coordinates (x, y, z) as determined by Equations (10) and (11).

**Figure 17:**
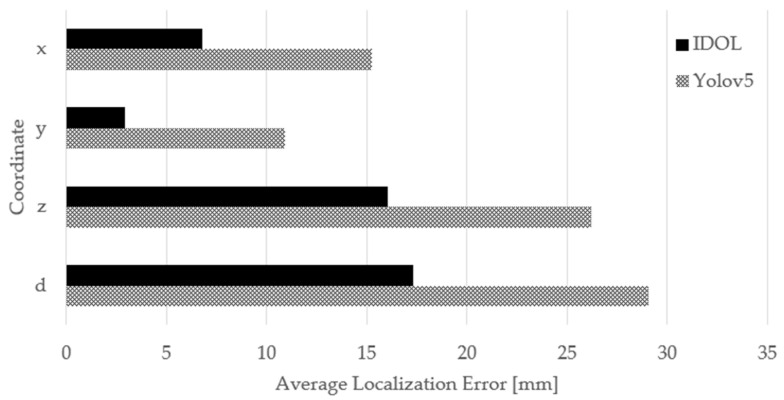
Average localization error of the IDOL and YOLOv5.

The object localization errors of the IDOL and YOLOv5 in depth (D) and (x, y, z) coordinates are compared and analyzed for each class of small and medium-sized indoor construction tools. Figure 17 displays the average localization error of the IDOL and YOLOv5 localization in 3D point cloud data.

The results indicate that the IDOL algorithm displays superior object localization performance compared to the YOLOv5 object detection model, resulting in a closer approximation to the ground truth coordinates. The average localization error of the 3D point cloud coordinates (x, y, z, D) of the IDOL algorithm was found to be less than 18 mm. These findings suggest that the object localization of the IDOL algorithm is more valid and accurate than that of the YOLOv5 object detection model.

Safety managers and supervisors face challenges in ensuring indoor construction site safety and visualization aids should be more precise and less error-prone in terms of 2D images and 3D point cloud simulations. A lower localization error in 3D point cloud localization results provides an improved visualization and a closer representation of the actual construction site when the tools are localized. Therefore, the IDOL algorithm offers an efficient and effective method for localizing small and medium-sized construction tools, thus improving safety management in indoor construction sites.

## 6. Conclusions

The improved discriminative object localization (IDOL) algorithm has been developed for small and medium-sized construction tools used in indoor construction sites. The Grad-CAM on the EfficientNet-B7 classification neural network process is applied to enhance safety management efficiency and effectiveness.

Evaluation of localization coordinates of objects obtained from ground truth, bounding box results of YOLOv5 object detection model, and Grad-CAM results of the IDOL algorithm demonstrated that the resulting coordinates of the IDOL algorithm have increased localization accuracy in 2D images by 31.7% and decreased the average localization error of depth coordinates by 40.4% compared to YOLOv5 detection model. While the results of YOLOv5 include incorrect coordinates within the bounding box, the IDOL utilizes Grad-CAM to concentrate on features of objects, thereby excluding most of the irrelevant coordinates that do not correspond to actual objects and producing localization coordinates that are closer to the ground truth segmentation. Thus, the proposed algorithm, the IDOL, was validated by experiment and proven to be a reliable and effective object localization method with high accuracy in producing 2D images and 3D coordinates.

The development of the IDOL algorithm provides accurate 3D localization of objects and assists with safety management. The proposed IDOL algorithm extends the target range of existing research studies on construction site object recognition. Integrating the actual construction site and its 3D simulation when mapping indoor construction sites could serve as a basis for future research on safety management by object recognition.

## Figures and Tables

**Table 1:** YOLOv5 models results on small and medium-sized construction tools [9]. (Reproduced with permission from Lee et al., KSCE Journal of Civil Engineering; published by Korean Society of Civil Engineers, 2023).

Model	Algorithm	Precision (%)	Recall (%)	mAP (%)	FPS
M1	YOLOv5x	81.2	73.2	69.1	58.82
M2	YOLOv5s	85.1	67.4	64.4	142.68

**Table 2:** Examples of 3D point cloud data and corresponding calculated depth value.

x	y	z	U	V	Depth (D)
0.373137	−0.00636	1.999704	1254	531	2.034229
0.379566	−0.00631	1.983881	1261	531	2.019875
0.383699	−0.00624	1.957031	1268	531	1.9943
0.349937	0.002966	2.029182	1232	538	2.059137

**Table 3:** Localization errors of the IDOL and YOLOv5 in (x, y, z) and (D) coordinates.

Experimental Cases		x			y			z			D		
		GT	IDOL	YOLOv5	GT	IDOL	YOLOv5	GT	IDOL	YOLOv5	GT	IDOL	YOLOv5
Bucket	location [mm]	16.32	10.85	22.43	7.82	3.52	−40.37	698.57	814.84	817.45	710.22	836.38	840.49
	error [mm]	-	5.47	6.11	-	4.30	48.19	-	116.27	118.87	-	126.15	130.27
Cutter	location [mm]	−3.55	−18.84	−25.77	77.38	27.82	6.69	1195.69	1114.31	1459.85	1209.44	1120.79	1487.39
	error [mm]	-	15.29	22.22	-	49.56	70.69	-	81.37	264.16	-	88.65	227.95
Drill	location [mm]	4.79	42.36	−371.58	100.12	122.62	−382.33	733.84	1201.57	2058.49	745.67	1220.27	2128.76
	error [mm]	-	37.58	376.37	-	22.51	482.45	-	467.74	1324.66	-	474.60	1383.09
Grinder	location [mm]	92.41	95.86	57.90	25.27	49.07	−67.19	485.21	576.78	736.33	501.40	597.39	761.93
	error [mm]	-	3.45	34.52	-	23.80	92.46	-	91.57	251.12	-	95.98	260.53
Hammer	location [mm]	155.35	−787.44	124.14	−1.78	84.17	−23.91	578.52	1842.20	1260.82	600.64	2008.39	1270.26
	error [mm]	-	157.19	754.39	-	30.77	77.30	-	288.03	294.54	-	314.30	423.83
Knife	location [mm]	155.35	157.60	−0.48	−1.787	−3.58	5.96	578.52	584.73	584.52	600.64	607.87	585.21
	error [mm]	-	2.24	155.83	-	1.80	7.74	-	6.21	6.00	-	7.24	15.43
Saw	location [mm]	−37.33	−26.52	103.86	−40.95	−42.33	−53.51	811.51	833.47	868.04	820.82	840.47	875.88
	error [mm]	-	10.81	141.19	-	2.28	12.56	-	21.96	56.53	-	19.65	55.06
Shovel	location [mm]	−289.39	33.92	71.30	−259.65	−155.28	−93.33	2397.31	1665.28	1960.29	2514.41	1677.67	1997.48
	error [mm]	-	323.30	360.69	-	104.36	166.31	-	732.03	437.02	-	836.74	519.93
Spanner	location [mm]	75.46	62.93	52.55	106.46	66.26	110.51	1238.92	1276.23	1243.61	1252.70	1280.65	1257.86
	error [mm]	-	12.53	22.91	-	40.20	4.05	-	37.31	4.68	-	27.94	5.16
Tacker	location [mm]	168.56	151.17	81.04	−51.00	−44.76	56.99	679.49	704.75	665.62	705.99	729.13	664.00
	error [mm]	-	17.39	87.52	-	6.24	107.99	-	25.26	23.87	-	23.14	41.99
Trowel	location [mm]	7.23	−55.76	23.76	32.47	−21.55	−32.53	531.88	573.61	682.54	538.36	578.16	731.31
	error [mm]	-	62.98	16.53	-	54.02	65.00	-	41.74	150.66	-	39.79	192.94
Wrench	location [mm]	−190.60	−209.14	−189.83	−14.97	−12.00	−192.32	1283.54	1267.65	1069.44	1300.28	1285.15	1109.89
	error [mm]	-	12.54	6.77	-	2.96	177.36	-	15.89	214.10	-	15.13	190.39
Average Localization Error		-	11.30	15.26	-	2.91	10.88	-	16.04	26.21	-	17.31	29.04

## Data Availability

The datasets of Lee [9] used during the current study are available in the Zenodo repository, https://doi.org/10.5281/zenodo.6530106 (accessed on 12 December 2022).
